# Climate‐Smart Bread With Cauliflower Leaf Powder: Enhancing Nutrition and Reducing Food System Waste and Carbon Footprint, Addressing Sensory Trade‐Offs and Improvement Opportunities

**DOI:** 10.1002/fsn3.71533

**Published:** 2026-02-13

**Authors:** Zeweter Abebe, Aklesia Haileyesus

**Affiliations:** ^1^ Center for Food Science, Addis Ababa University Addis Ababa Ethiopia

**Keywords:** antinutritional factors, cauliflower leaf powder, climate‐smart food system, food waste reduction, nutritional enhancement

## Abstract

*Brassica oleracea var. botrytis* or cauliflower is enjoyed by many around the world. However, its nutrient‐rich leaves are commonly discarded; its nutrient‐rich leaves are thrown away, and we waste food and miss out on edible resources. This study looked at the nutritional and antinutritional profile of cauliflower leaves after boiling, blanching, or fermentation. It also looked at their potential to boost nutrients in wheat bread. The processed leaves were dried, powdered, and mixed with wheat flour at levels of 1%–9% to make bread. The bread samples were tested for nutritional content, antinutrients, functional properties, and taste. Environmental impact was estimated by comparing water and carbon footprint. Fermentation improved the nutritional quality of cauliflower leaf powder (CLP), increased mineral content and energy, and decreased antinutrients. This was done without affecting protein and fiber content. Adding CLP to bread increased protein and fiber content and enhanced iron and zinc bioavailability. Sensory acceptability was good up to 5% substitution. Partial replacement of wheat flour with CLP also gave environmental benefits by reducing water use and carbon emissions. These results show that CLP is a functional, climate‐friendly, and nutrient‐rich ingredient for bread production. It's a practical solution to postharvest loss and dietary issues and also highlights the importance of ensuring that nutritional improvements are acceptable to consumers.

## Graphical abstract

This study explored the use of fermented cauliflower leaf powder (CLP) as a nutrient‐rich, sustainable wheat flour substitute in bread. Fermentation improved the nutritional value of cauliflower leaves while reducing antinutrients. Bread enriched with up to 9% CLP showed higher protein and fiber, better mineral bioavailability, and lower water and carbon footprints, demonstrating CLP's potential as a functional ingredient that enhances nutrition and reduces environmental impact.

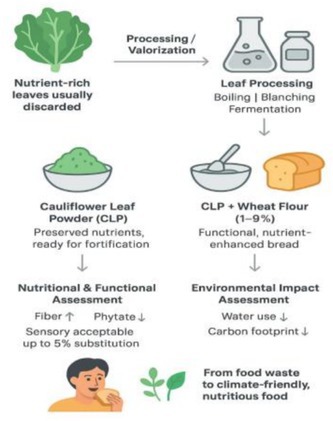

## 1 Introduction

The world still faces malnutrition as one of the biggest health concerns, despite the fact that a huge variety of edible plants are available (Von Grebmer et al. 2014). The nonuse of nutritious plant parts is one of the causes, which is very common during the marketing and processing of these plants. The nutrients that are lost in this way are not only a cause of nutrient deficiencies, but also contribute to waste in the food system, hence, delaying the attainment of the Sustainable Development Goals (SDGs), especially SDG 2 (Zero Hunger) and SDG 12 (Responsible Consumption and Production) (Colglazier 2015).

Cauliflower ( *Brassica oleracea* var. *botrytis* ) is one of the top green vegetable crops on a global scale, and its inclusion in the Ethiopian diet is common (FAO 2020). Cauliflower, however, has one of the highest waste indices along with limited commercial use (Kaur et al. 2022). This results in the throwing away of 70%–80% of the plant parts, such as the leaves and stems, after the white curd has been taken out. These leaves, however, are now scientifically proven to be very rich in protein, fiber, and minerals, thereby providing the ability to improve the nutritional status of under five‐year‐olds (Singh et al. 2019). In addition, cauliflower leaves have been considered as potential ingredients for enhancing the nutritional content of various staple foods that are usually consumed (Catana et al. 2024; Tukassar et al. 2023). The good news from research is that these leaves can be utilized if the effects of antinutritional factors, such as oxalates and phytates, along with possible undesirable sensory attributes, are mitigated.

The use of boiling, blanching, or fermentation as processing methods can be extremely helpful in reducing the antinutrients, improving nutrient bioavailability (Samtiya et al. 2020), and consumer acceptance (Akomea‐Frempong et al. 2021), depending on the demand. This is particularly pivotal when preparing foods for vulnerable groups, such as pregnant and lactating women, for whom nutrient density and safety are crucial. Also, repurposing vegetable by‐products by incorporating them into staple foods offers a practical approach to address nutrient deficiencies while promoting environmental sustainability. A major methodological advantage of this study is its holistic approach, combining food processing techniques, assessment of nutritional quality and antinutrient content, evaluation of sensory properties, and consideration of environmental impacts. This allows for a thorough assessment of the feasibility and benefits of incorporating CLP into bread as a nutrient‐rich, climate‐conscious food product. Based on this premise, adding cauliflower leaf powder (CLP) to dietary staples like bread could support dietary diversification, enhance nutrient density, and ensure climate‐ and resource‐conscious operations.

Previous studies have explored adding vegetable leaf powders, such as *Telfairia occidentalis* , *Amaranthus viridis* , and *Solanum macrocarpon* , to bread to improve nutritional value. However, the use of cauliflower leaves, along with their processing effects on nutritional and functional properties, has not been evaluated (Odunlade et al. 2017). There is limited information on how different processing methods affect the nutritional and antinutritional profiles of cauliflower leaves or how their inclusion impacts nutrition, health, and consumer acceptability (Elhassaneen et al. 2024; Mogra et al. 2012). Similarly, the climate‐smart benefits of cauliflower leaves, such as reducing carbon dioxide emissions by diverting waste and conserving water, have not been well documented. This research seeks to fill these gaps by investigating fermented cauliflower leaf powder as a value added ingredient in bread. Furthermore, there is limited evidence regarding how different processing methods influence the nutritional and antinutritional composition of cauliflower leaves, their incorporation into staple foods, and associated climate‐smart benefits, including waste diversion and water savings.

Therefore, this study aimed to determine the nutritional and antinutritional composition of cauliflower leaves subjected to boiling, blanching, and fermentation. It also evaluated the acceptability of CLP as a nutrient enhancer in wheat bread formulations. Finally, it examined the potential of CLP to reduce glycemic load and contribute to food waste reduction, water savings, and mitigation of carbon footprint. Based on the literature, it was hypothesized that fermentation would best enhance the nutritional profile of CLP while reducing antinutrients, and that its incorporation into bread would produce a nutritionally and environmentally improved product without compromising sensory acceptability.

## 2 Materials and Methods

### 2.1 Sample Collection

The cauliflower leaf samples were collected early morning from randomly selected vegetable markets established by Addis Ababa city administration. The vegetable markets in the city were identified from Addis Ababa city trade and industry bureau registry. One‐third of the vegetable shops within the selected markets were identified for sample collection using a lottery method.

Vegetable shops that were selling cauliflower under shade were selected for the study. Cauliflowers that were fresh‐looking and free from bruises were transported to the laboratory after covering them with plastic bags followed by immediate separation of the white curd from the leaves and cleaning under running water ~5 L/kg. Only cauliflower leaves from cauliflowers available at the selected markets were collected, as these reflect the portion of leaves that typically enter the consumer and processing chain. Leaves left at the farm or discarded before reaching the market were not included.

### 2.2 Pre‐Processing and Leaf Powder Preparation

The cleaned leaves were randomly assigned to salt fermentation, boiling, and blanching processes, then the remaining leaves were allocated to the control group without processing. The fermentation of cauliflower leaves was carried out for 8 days in a plastic jar with 30 g of salt per 1000 g of leaves. This duration aligns with best‐practice guidelines for vegetable fermentation at ambient temperatures (~25°C) which indicate that lactic acid fermentation can reach sufficient acidity within 7–10 days while maintaining product safety and quality (McIntyre et al. 2024). The leaves were fully submerged to ensure anaerobic conditions, as recommended for optimal LAB growth.

Similarly, to boil the leaves, they were left in boiling water for 5 min, allowing them to soften. Blanching was done by dipping the leaves in boiled water for 2 min followed by immediate immersion in cold water. Next, the processed leaves were dried in an oven (Genlab. model OV/125/SS/F/DIG/A) with uniform heat distribution and maintained at 45°C. It took 16 h for the boiled, blanched, and fermented leaves to dry, while it took only 14 h for raw leaves to dry. The dried cauliflower leaves were ground into a fine powder using a Xian Siway Scientific high‐speed sample miller (model ZN‐08) and passed through a 60‐mesh sieve (≈250 μm) to ensure uniform particle size for subsequent analysis.

### 2.3 Formulation of Wheat Flour and Cauliflower Leaves

The levels of CLP added to wheat flour (0%–9%) were determined based on previous studies that incorporated green leafy vegetable powders into bread (Famuwagun et al. 2016; Odunlade et al. 2017), ensuring both nutritional enhancement and acceptable sensory quality. The powders were thoroughly mixed with wheat flour using a flour mixer.

The different flour formulations were homogenized with yeast (4 g yeast per 100 g of flour), and the dough was allowed to ferment for 4 h at an ambient temperature of approximately 27°C and a relative humidity of 60%, covered with a damp cloth to prevent moisture loss and ensure uniform yeast activity. Subsequently, baking was carried out in an oven (Vigor, model 3 decks) at 200°C for approximately 35 min, until the loaves developed a golden‐brown crust and exhibited a hollow resonance upon tapping.

### 2.4 Determination of Proximate Composition

Crude protein content was determined using Kjeldahl method using 6.25 as a conversion factor (AOAC 2005). Moisture content and crude fiber were analyzed using standard methods (AOAC 2005). Fat and Ash content were determined by soxhlet extraction and dry ashing methods respectively (AOAC 2005).

### 2.5 Determination of Antinutritional Factors

Oxalate and phytate content of cauliflower leaf under different processing methods were determined using titration method (Abdel Moemin 2014) and UV–Vis spectroscopy (AOAC method) respectively (AOAC 2005).

### 2.6 Determination of Gluten Content and Flour Functional Property

Gluten and functional properties of breads made from the different CLP proportions were determined using hand washing AACC (2000) and Aremu et al. (2007) respective methods.

### 2.7 Sensory Evaluation

The bread samples F1 to F6 were presented for sensory evaluation to ten panelists aged between 25 and 32 years, consisting of 5 males and 5 females. All the panelists were students of Food Science and Nutrition, had some experience in sensory evaluation, and regularly consumed bread products. Assessment was done using a hedonic scale with nine points, where 1 = dislike extremely, 2 = dislike very much, 3 = dislike moderately, 4 = dislike slightly, 5 = neither like nor dislike, 6 = like slightly, 7 = like moderately, 8 = like very much, and 9 = like extremely. Before starting the actual assessment, the hedonic scale was explained to the panelists. Each panelist conducted the test in a private, comfortable, quiet booth.

### 2.8 Practical Impact Estimation Methodology

To assess the real‐world nutritional and environmental benefits of substituting wheat flour with CLP, a series of calculations were performed to quantify impact per 100 g and per 1000 kg (1 ton) of bread. The calculations focused on five major areas: protein and fiber contribution, antinutrient reduction, glycemic load (GL), carbon footprint, and water savings. All calculations were based on standard reference values and assumptions documented in the literature, as outlined below.

#### 2.8.1 Protein Contribution

The additional protein provided by the enriched bread (F1: 9% CLP substitution) compared to the control (F6: 100% wheat flour) was calculated per 100 g of bread. The contribution to the Recommended Nutrient Intake (RNI) for pregnant and lactating women was estimated using FAO/WHO guidelines, which recommend a maximum daily protein intake of 58 g for pregnant women and 63 g for lactating mothers (FAO/WHO/UNU 2007). With 2.85 g of added protein from CLP‐enriched bread, the percentage contribution to the RNI was calculated using the following formula:

%RNI=Protein addedper100gbread÷RNI

#### 2.8.2 Dietary Fiber Contribution

The additional fiber intake of +2.75 g per 100 g bread was compared to the adequate intake (AI) for dietary fiber in women, which is 25 g/day (EFSA 2010). Thus, the enriched bread contribution to daily fiber needs per 100 g consumed was calculated using the following formula:

%AIcontribution=Additional fiberper100g÷AI

#### 2.8.3 Antinutrient Reduction: Phytate

Phytate to iron and phytate to zinc molar ratios were calculated to estimate mineral bioavailability using the formula:

$$\text{Molar Ratio}=\left(\text{Phytate}\;\left(\mathrm{mg}\right)/660\right)÷\left(\text{Mineral}\;\left(\mathrm{mg}\right)/\text{Atomic Weight}\right).$$

where 660 is the molecular weight of phytic acid (C_6_H_18_O_24_P_6_), Atomic weights used were 55.85 and 65.38 g/mol for iron and zinc.

In the control bread (F6), phytate:iron and phytate:zinc molar ratios were 0.046 and 0.076, respectively. In the enriched bread (F1), the ratios were 0.015 for iron and 0.028 for zinc. All values were well below the critical thresholds (1 for iron and 15 for zinc), indicating good mineral bioavailability. The reduced phytate content in the enriched bread, likely due to fermentation and substitution with CLP, may enhance mineral absorption, consistent with findings by Hurrell (2004).

#### 2.8.4 Glycemic Load (GL) Reduction

Glycemic load (GL) was estimated to evaluate the potential glycemic response associated with consumption of the bread samples. GL provides a more accurate assessment of carbohydrate quality than glycemic index (GI) alone by accounting for the actual amount of carbohydrate in a given portion.

GL was calculated using the following formula:

$$\mathrm{GL}=\left(\text{Available Carbohydrate}\;\left(g/100\;g\right)\times \text{Glycemic Index}\right)/100$$

Available carbohydrate content was derived from proximate analysis by subtracting crude fiber from total carbohydrates. A GI value of 70 was assumed for both samples, based on reference values for refined wheat bread obtained from the University of Sydney (2021). This assumption enabled standardized comparison between control and enriched formulations.

To estimate the relative reduction in glycemic impact due to formulation changes, the percentage change in GL between control and enriched bread samples was also calculated using the following formula:

$$\%\mathrm{GL}\;\text{Reduction}=\left(\mathrm{GL}-\text{control}-\mathrm{GL}-\text{enriched}\right)/\mathrm{GL}-\text{control}\times 100$$

These calculations allowed for the assessment of how changes in available carbohydrate content particularly those influenced by the addition of CLP may affect the potential glycemic response of the bread products.

#### 2.8.5 Carbon Footprint Reduction

The carbon footprint (CF) of the bread formulations was estimated to evaluate the environmental impact of substituting wheat flour with cauliflower leaf powder (CLP). Emission factors were sourced from existing literature: 1.1 kg CO_2_e/kg for wheat flour (Espinoza Orias et al. 2011) and 0.18 kg CO_2_e/kg for CLP. The emission factor for cauliflower leaf powder (0.18 kg CO_2_e/kg) was approximated from the generalized category of low‐impact vegetables reported by Poore and Nemecek (2018), as specific life cycle data for cauliflower leaves are not available in the literature.

The carbon footprint was calculated using the formula:

$$\mathrm{CF}=\left(\text{Amount of ingredient used in}\;\mathrm{kg}\right)\times \left(\text{Emission factor in}\;\mathrm{kg}\;{\mathrm{CO}}_{2}e/\mathrm{kg}\right)$$

Calculations were performed per 1000 kg of bread to standardize comparisons between the control (100% wheat flour) and enriched bread (91% wheat flour, 9% CLP). The relative reduction in carbon emissions due to ingredient substitution was calculated as:

$$\%\text{Reduction}=\left(\left(\mathrm{CF}\_\text{control}-\mathrm{CF}\_\text{enriched}\right)/\mathrm{CF}\_\text{control}\right)\times 100$$

To estimate product‐level impact, the reduction was also scaled to a 100 g serving size.

#### 2.8.6 Cauliflower Leaf Biomass Repurposing

The quantity of fresh cauliflower leaf required to produce the CLP used in the enriched bread was estimated to assess the potential for agricultural waste valorization. The moisture content of fresh leaves was assumed to be approximately 88%, based on literature values (Furia et al. 2025), while the drying yield (12%) and cleaning efficiency (90%) were also assumed for subsequent calculations. The required amount of fresh leaf per kilogram of CLP was calculated as:

$$\begin{array}{c} \text{Fresh leaf required}\;\mathrm{per}\;\mathrm{kg}\;\mathrm{CLP}=1/\left(\text{Drying yield}\times \text{Cleaning efficiency}\right)\\ =1/\left(0.12\times 0.90\right)\approx 8.3\;\mathrm{kg} \end{array}$$

Based on the substitution rate (9% CLP, or 90 kg per 1000 kg of bread), the total fresh biomass required was estimated using:

Total fresh biomass=CLPrequired×Fresh leafperkgCLP

#### 2.8.7 Water Footprint Estimation

The water footprint of the bread formulations was assessed to evaluate the potential reduction in freshwater use resulting from the partial substitution of wheat flour with CLP. The water footprint of wheat flour was taken as 1827 L/kg, based on global average estimates by Mekonnen and Hoekstra (2011).

Cauliflower leaves were treated as an agricultural by‐product with no additional irrigation requirements. Therefore, only the water used during postharvest processing was considered, primarily for cleaning. The volume of water required for processing was estimated at 5 L per kg of fresh leaf, based on standard washing procedures.

To estimate the water used in CLP processing, the total amount of fresh cauliflower leaf required for CLP production was used (as determined in Section 2.8.6). The water required for washing was calculated as:

$$\text{Water for processing}\;\left(L\right)=\text{Fresh leaf biomass}\;\left(\mathrm{kg}\right)\times 5\;L/\mathrm{kg}$$

In contrast, the water that would have been used to produce the equivalent amount of wheat flour replaced was calculated as:

$$\text{Water footprint of wheat flour}\;\left(L\right)=\text{Wheat flour replaced}\;\left(\mathrm{kg}\right)\times 1827\;L/\mathrm{kg}$$

The net water saving was calculated as the difference between the water avoided from wheat flour production and the water used for CLP processing:

$$\begin{array}{c} \mathrm{Net}\;\text{water saving}\;\left(L\right)=\text{Water avoided}\;from\;\left(\text{wheat flour}\right)\;production\\ -\text{Water used}\;\left(\mathrm{CLP}\;\text{processing}\right)\approx 16,398\;L\;\mathrm{per}\;\;\mathrm{100 kg}\;\text{bread}, \end{array}$$

corresponding to ~9% reduction relative to the water footprint of an equivalent mass of wheat flour, and this value was used for graphical presentation.

### 2.9 Data Analysis

Data were analyzed using SPSS version 20. Analysis of variance (ANOVA) was performed to determine significant differences among means. The assumptions of ANOVA were checked prior to analysis using the Shapiro–Wilk test for normality and Levene's test for homogeneity of variances, and the data met these assumptions.

### 2.10 Ethical Clearance

Ethical clearance was granted by the Institutional Review Board of the College of Natural and Computational Sciences (Minute No. IBR/05/2015/2023).

## 3 Results

### 3.1 Proximate Composition of CLP

Processing markedly altered the proximate composition of cauliflower leaves (Table 1). Fermentation resulted in the greatest moisture reduction, increased ash content, and relatively preserved fat and energy levels, while raw leaves maintained the highest protein and fiber, which decreased following thermal treatments, particularly boiling, accompanied by higher carbohydrate content.

**TABLE 1:** Proximate composition, energy, oxalate and phytate contents of cauliflower leaves under different processing methods.

Sample type	Moisture (g/100 g)	Crude protein (g/100 g)	Crude fat (g/100 g)	Crude fiber (g/100 g)	Total ash (g/100 g)	Carbohydrate (g/100 g)	Energy (kcal)	Oxalate (mg/100 g)	Phytate (mg/100 g)
Raw	11.95 ± 0.63^a^	32.45 ± 0.35^a^	1.75 ± 0.35^a^	23.5 ± 0.70^a^	22.8 ± 0.28^a^	7.85 ± 0.35^a^	173.76 ± 0.01^a^	2.74 ± 0.13ᵃ	1.15 ± 0.02ᵃ
Boiled	11.37 ± 0^a^	26.21 ± 0.30^b^	1.25 ± 0.35^b^	12.5 ± 0.70^b^	24.4 ± 0.90^a^	24.07 ± 0.85^b^	212.37 ± 0.96^b^	1.27 ± 0.08ᵇ	0.96 ± 0.17ᵇ
Blanched	8.02 ± 0.11^b^	29.25 ± 0.25^c^	1.15 ± 0.35^b^	9.5 ± 0.70^c^	20.9 ± 0.84^b^	31.18 ± 0.36^c^	252.07 ± 0.86^c^	1.93 ± 0.13ᵇ	0.82 ± 0.31ᶜ
Fermented	4.33 ± 0.19^c^	20.13 ± 0.24^d^	1.75 ± 0.35^a^	21.65 ± 0.49^d^	42.74 ± 0.4^c^	9.4 ± 0.49^d^	267.21 ± 0.19^d^	0.78 ± 0.22ᶜ	0.51 ± 0.07ᵈ
*p*‐value	< 0.001	< 0.001	0.030	< 0.001	< 0.001	< 0.001	< 0.001	< 0.001	0.002

*Note:* Processing methods included raw (unprocessed), boiled, blanched, and fermented cauliflower leaves. Values are expressed as g/100 g on a dry‐weight basis, unless otherwise stated, and are presented as mean ± standard deviation (*n* = 3). Each value represents the mean of three technical replicates obtained from the same sample batch. Different superscript letters (a–d) within the same column indicate statistically significant differences among processing methods, as determined by one‐way ANOVA (*p* < 0.05).

### 3.2 Oxalate and Phytate Contents of CLP

Processing significantly affected antinutrient content of CLP. Raw samples had the highest oxalate and phytate levels, which declined following thermal and microbial treatments. Fermentation led to the greatest reduction, followed by blanching and boiling, indicating that both thermal and microbial processing effectively lower antinutritional factors (Table 1).

### 3.3 Proximate Composition of Bread Products

The addition of CLP into bread formulations significantly influenced proximate composition and energy content (Table 2). Crude protein content increased progressively with higher CLP substitution, with F1 showing the highest protein level (*p* < 0.05). Crude fiber and ash contents also increased in CLP‐enriched breads, particularly in F1 and F2, indicating improved dietary fiber and mineral contribution. Conversely, carbohydrate content decreased with greater CLP addition, reflecting the dilution of starchy wheat flour.

**TABLE 2:** Proximate composition and energy content of bread formulated with varying proportions of cauliflower leaf powder and wheat flour (weight basis).

Sample code	Moisture (g/100 g)	Crude protein (g/100 g)	Crude fat (g/100 g)	Crude fiber (g/100 g)	Total ash (g/100 g)	Carbohydrate (g/100 g)	Energy (kcal)
F1	13.78 ± 0.31ᵃ	10.76 ± 0.12ᵃ	1.00 ± 0.00ᵃᵇ	4.50 ± 0.70ᵃ	1.22 ± 0.007ᵃ	68.83 ± 0.89ᵃ	327.40 ± 0.07ᵃ
F2	13.47 ± 0.10ᵃᵇ	10.40 ± 0.00ᵃ	2.25 ± 0.35ᶜ	3.75 ± 0.35ᵃᵇ	3.09 ± 0.16ᵇ	67.03 ± 0.97ᵇ	329.97 ± 0.72ᵃ
F3	13.10 ± 0.07ᵇ	10.34 ± 0.22ᵃ	1.25 ± 0.35ᵃᵇ	2.75 ± 0.35ᵇᶜ	3.18 ± 0.26ᵇ	69.37 ± 0.29ᵃ	330.11 ± 0.11ᵃ
F4	13.35 ± 0.50ᵃᵇ	9.71 ± 0.12ᵇ	1.25 ± 0.35ᵃᵇ	2.00 ± 0.70ᶜ	3.29 ± 0.11ᵇ	70.39 ± 0.54ᵃ	331.67 ± 0.49ᵃ
F5	13.96 ± 0.05ᵃ	9.01 ± 0.37ᶜ	1.75 ± 0.35ᵇᶜ	2.00 ± 0.00ᶜ	1.02 ± 0.26ᵃ	72.26 ± 0.30ᶜ	340.85 ± 0.49ᵇ
F6 (control)	13.93 ± 0.01ᵃ	7.91 ± 0.30ᵈ	0.75 ± 0.35ᵃ	1.75 ± 0.35ᶜ	0.94 ± 0.12ᵃ	74.72 ± 0.51ᵈ	337.29 ± 0.07ᵇ
*p*‐value	0.066	< 0.000	0.029	0.006	< 0.000	< 0.000	0.002

*Note:* Values are expressed as mean ± standard deviation (*n* = 3) and reported on a dry‐weight basis. Significant differences among formulations were determined using one‐way ANOVA (*p* < 0.05); different superscript letters (a–d) within a column indicate statistically significant differences among sample means. F1–F5 represent composite breads containing increasing levels of cauliflower leaf powder (CLP) (F1 = 3%, F2 = 5%, F3 = 7%, F4 = 9%, F5 = 11% CLP), while F6 represents the control formulation prepared with 100% wheat flour.

Energy values remained relatively stable across samples, though F5 and F6 had slightly higher caloric content due to increased carbohydrate levels (Table 2). Moisture and fat content showed minimal variation, with no consistent trend across formulations. Overall, CLP‐enriched breads demonstrated enhanced nutritional profiles, especially in protein, fiber, and ash content, without compromising energy density.

### 3.4 Water Absorption Capacity and Gluten Content

The water absorption capacity and the gluten content of breads with varying levels of CLP are presented in Table S1. Significant differences among formulations were determined by ANOVA (*p* < 0.05). The results showed variation in water absorption capacity, wet gluten content, and wet gluten‐to‐protein ratio.

### 3.5 Sensory Evaluation

**FIGURE 1:**
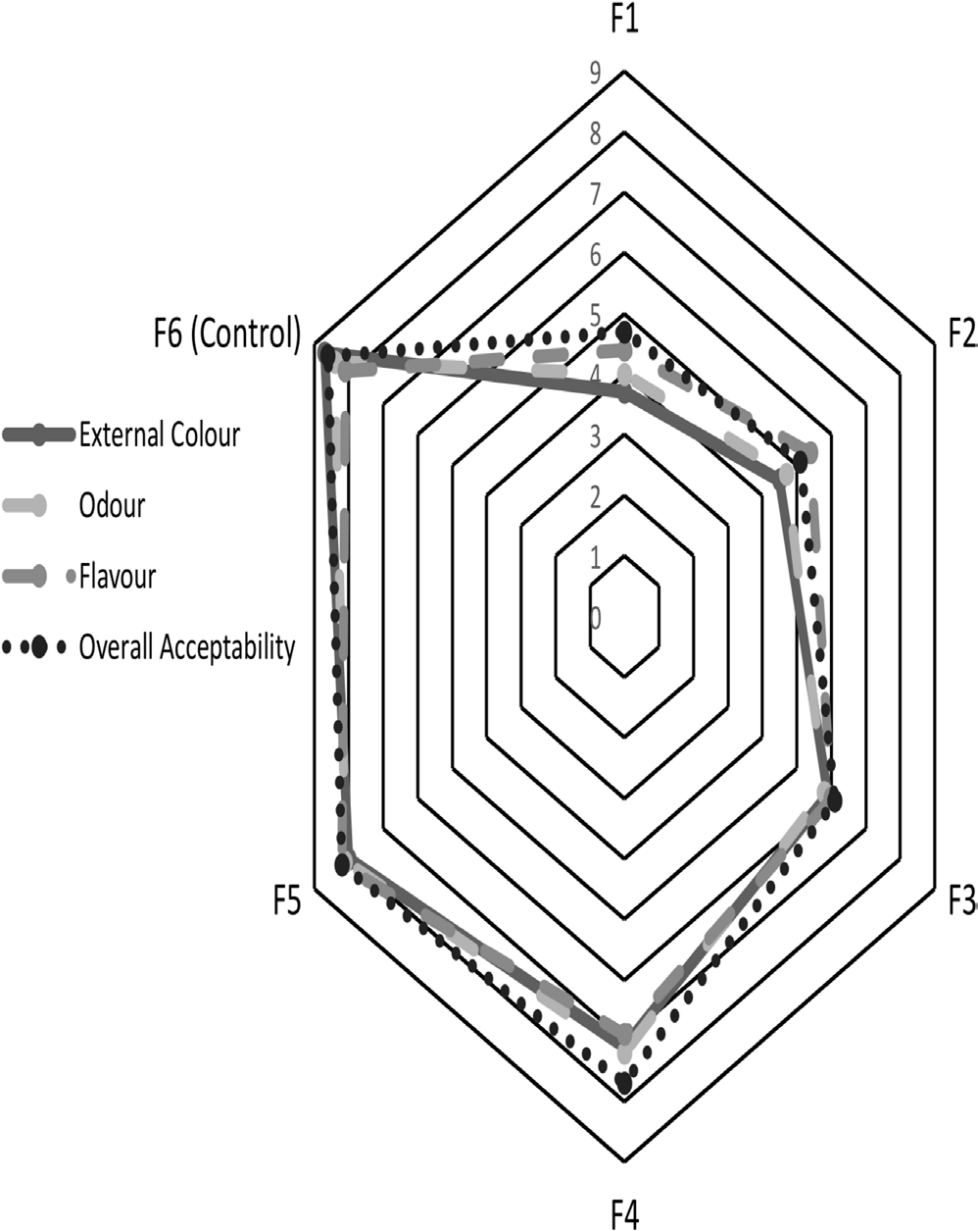
Spider plot of mean sensory scores of bread samples (F1–F6) for external color, odor, flavor, and overall acceptability. The spider plot displays mean sensory scores for each bread sample as evaluated by 10 panelists using a 9‐point hedonic scale (1 = dislike extremely; 9 = like extremely). Each point represents the average score for the respective attribute (external color, odor, flavor, and overall acceptability). No statistical comparisons were performed.

The sensory evaluation revealed that panelists' acceptance of bread samples improved with decreasing levels of CLP (Figure 1). The control sample (F6) received the highest scores across all attributes, particularly for external color (8.7), odor (8.4), and overall acceptability (8.6).

Among the enriched samples, F5 was most preferred, scoring above 8 in all categories. Samples F1 and F2 received the lowest ratings, especially in external color and flavor, indicating limited consumer appeal at higher CLP substitution levels (Figure 1). Overall, moderate inclusion of CLP (as in F4 and F5) maintained favorable sensory qualities while enhancing nutritional value.

### 3.6 Nutritional and Environmental Impact of Bread Enriched With Cauliflower Leaf Powder

Adding 9% CLP to wheat bread resulted in an increase in protein content and dietary fiber, reflecting meaningful nutritional enhancement. These values represent estimated impacts rather than statistically tested outcomes. This increase in protein content is equivalent to 5% of the recommended nutrient intake (RNI) for pregnant and lactating women, and dietary fiber increased from 1.75 to 4.50 g per 100 g, contributing over 10% of the daily recommended intake.

**FIGURE 2:**
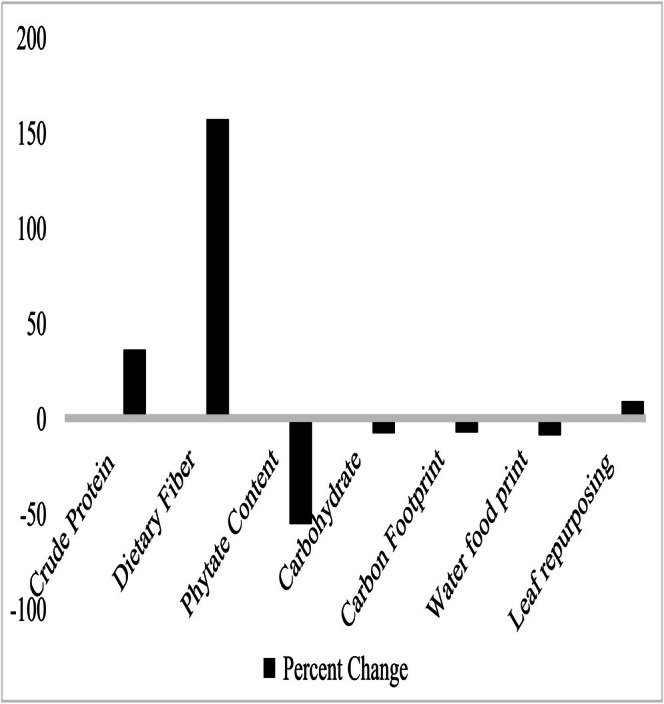
Percentage change in nutritional and environmental indicators following 9% cauliflower leaf powder enrichment of wheat bread (F1 vs. Control F6). Values represent estimated impacts rather than statistically tested outcomes.

Phytate content was substantially reduced following fermentation of the cauliflower leaf powder, indicating an improvement in mineral bioavailability. Bread enriched with 9% CLP also showed a moderate decrease in carbohydrate concentration (Figure 2), which corresponds to a lower estimated glycemic load compared to the control.

In terms of environmental impact, enrichment reduced the product's carbon footprint, contributed to meaningful vegetable waste diversion, and resulted in water savings due to the replacement of irrigated wheat flour with cauliflower leaves. Full numerical values are presented in Table 3.

**TABLE 3:** Practical significance of nutritional and environmental changes in cauliflower‐leaf–enriched bread (F1, 9% CLP) compared to control (F6).

Impact area	Practical significance
Protein	May contribute approximately 5% of the daily RNI for pregnant and lactating women,^a^ supporting improved protein adequacy.
Dietary fiber	Provides more than 10% of the recommended daily fiber intake,^b^ which may assist digestive health.
Reduced phytate	Fermentation lowered phytate by ~55%, which can improve iron and zinc bioavailability by ~15%–20%,^c^, ^d^ potentially supporting micronutrient absorption. Gibson et al. 2006
Lower carbohydrates/glycemic load	A reduction of ~7.9% carbohydrates corresponds to an estimated ~11.8% decrease in glycemic load,^e^ beneficial for blood glucose management.
Lower carbon footprint	Producing enriched bread results in ~7.5% less CO_2_e, equivalent to saving ~8 g CO_2_e per 100 g of bread.^e^
Leaf repurposing	Utilizes ~9 kg cauliflower leaf per 100 kg of bread, preventing ~833 kg of vegetable waste per 1 ton of bread (calculated from production yield).
Water savings	Replacing 9% wheat flour avoids additional irrigation, saving ~16 L per 100 g of bread,^f^ equivalent to ~160,000 L saved per 1 ton.

*Note:* Values represent estimated nutritional and environmental impacts calculated per 100 g of bread based on observed compositional differences and literature‐derived assumptions. Environmental indicators include estimated vegetable waste reduction and potential CO_2_‐equivalent savings associated with cauliflower leaf utilization. No statistical testing was performed, and results should be interpreted as indicative estimates rather than inferential outcomes.

^a^ FAO/WHO/UNU (2007).

^b^ EFSA (2010).

^c^ Hurrell (2004).

^d^ Gibson et al. (2006).

^e^ Foster‐Powell et al. (2002).

^f^ Furia et al. (2025).

## 4 Discussion

### 4.1 Effect of Processing on CLP Composition

This study demonstrates the potential of cauliflower leaf powder (CLP) as a sustainable nutrient enhancer for bread, contributing to the broader goal of developing climate‐smart food products. The findings indicate improvements in bread nutritional quality, reductions in environmental impact, and sensory challenges associated with higher substitution levels of CLP. A major strength of this research lies in its holistic approach, combining food processing, nutritional assessment, antinutrient reduction, sensory evaluation, and environmental sustainability to comprehensively evaluate the feasibility and benefits of CLP‐enriched bread.

Fermentation markedly altered the proximate composition of CLP. Moisture content decreased and energy density increased following the process, while fat content remained unchanged. Ash content increased, indicating enhanced mineral concentration. These results align with previous reports showing that fermentation improves nutrient bioavailability through microbial degradation of antinutritional factors (Sun et al. 2024; Jeyakumar and Lawrence 2022). Conversely, boiling led to a significant reduction in protein and fiber, likely due to heat‐induced denaturation and leaching, while concentrating carbohydrate content (Dewangani et al. 2021; Arias‐Rico et al. 2020). Overall, fermentation appears to be the most beneficial method for retaining core nutrients and improving the functional quality of plant‐based food sources.

### 4.2 Nutritional and Functional Properties of Enriched Bread

Incorporating CLP into bread resulted in increases in crude protein, crude fiber, and ash content, particularly at higher substitution levels. The 9% CLP formulation (F1) showed a 36% increase in protein and a 157% increase in fiber compared to the control. Such nutritional gains are particularly relevant for vulnerable populations, including pregnant and lactating women, who have elevated protein and fiber requirements (Billeaud et al. 2021). Additionally, reductions in carbohydrate content and phytate levels in enriched breads correspond to lower estimated glycemic load and increased mineral bioavailability, particularly for iron and zinc, which are often limited in plant‐based diets due to phytate chelation (Kumar et al. 2020; Zhang et al. 2021).

Fermentation was the most effective in reducing oxalates and phytates, achieving over 70% and 50% reductions, respectively. While boiling and blanching also decreased these compounds, the effects were more modest. These findings support previous studies demonstrating that fermentation not only preserves nutrients but also mitigates mineral absorption inhibitors, reinforcing its role as a climate‐smart and health‐promoting food processing method (Gupta et al. 2015).

CLP addition also affected functional properties of the bread, including water absorption and gluten content. Increasing CLP inclusion decreased the gluten‐to‐protein ratio, indicating that the added protein does not contribute to the gluten network. This can affect dough rheology and bread texture; however, breads with up to 6% CLP maintained acceptable sensory profiles. It is important to acknowledge the limitations of the study. The sensory evaluation was conducted with a small panel of 10 participants, and only their age was recorded; therefore, the demographic diversity of the panel could not be characterized, and broader consumer acceptability and market potential cannot be fully generalized from these results. Moreover, the experiments were performed under controlled laboratory conditions rather than industrial‐scale settings, and variability in raw material characteristics may influence reproducibility.

Future studies involving larger, demographically diverse consumer panels and pilot‐ or industrial‐scale baking trials are recommended to validate the findings and improve generalizability. This suggests that formulation adjustments, such as the use of gluten enhancers or dough conditioners, could allow higher CLP inclusion without compromising product quality (Dai and Tyl 2021; Hathorn et al. 2008).

### 4.3 Sensory Profile and Consumer Acceptability

Higher enrichment levels negatively affected consumer acceptability, with breads containing 9% or higher CLP being less preferred, consistent with prior studies (Chauhan and Inteli 2015). This was likely due to darker color, vegetal flavor, and altered crumb texture. Breads with moderate inclusion (3%–6% CLP) maintained high sensory scores, demonstrating a functional balance between nutritional improvement and consumer acceptability. The most preferred enriched bread (F5) scored above 8.0 across all sensory attributes, highlighting the importance of optimizing inclusion levels and exploring strategies such as pretreatment, flavor‐masking agents, or consumer education campaigns to enhance marketability of climate‐smart breads (Feng et al. 2018; Shen 2024; Okonkwo et al. 2024; Bryden et al. 2024; Lee et al. 2009).

### 4.4 Environmental Benefits and Sustainability

Further, CLP inclusion provided clear environmental benefits. Replacing 9% of wheat flour with CLP decreased the carbon footprint by approximately 7.5% and saved 16 L of water per 100 g of bread. At industrial scales, these savings are substantial, reflecting the lower resource intensity of repurposing vegetable by‐products compared to cultivating staple grains. Additionally, using CLP diverted over 800 kg of vegetable waste per ton of bread, promoting food system circularity and supporting global strategies to reduce agro‐waste and greenhouse gas emissions (Rosa and Gabrielli 2023; Koul et al. 2022). A major strength of this study is the detailed calculations presented in Section 2.8 and summarized in Table 3, which translate laboratory findings into tangible, real‐world impacts, such as liters of water saved per ton of bread. These data provide a practical tool for policymakers and industry stakeholders to assess the relevance and scalability of such food innovations.

## 5 Conclusion

Fermentation is the most effective processing method to enhance the nutrient composition of CLP and reduce antinutritional factors. These findings support fermentation as a practical preprocessing option for incorporating cauliflower leaf powder into cereal‐based foods. CLP has the potential to serve as a sustainable bread nutrient enhancer and may be applied in bakery product reformulation to improve health outcomes by lowering the estimated glycemic load. Furthermore, the study's estimations suggest that CLP incorporation can reduce the environmental impact of bread production through decreased resource use, lower carbon and water footprints, and diversion of vegetable waste via leaf valorization, highlighting its relevance for climate‐smart food production strategies. To balance nutritional benefits with consumer acceptability, strategies such as pretreatment, flavor masking, and consumer education should be considered when translating these findings into product development and scale‐up. Overall, CLP‐enriched bread represents a promising approach for advancing climate‐smart, circular food systems that support both health and environmental sustainability.

## Author Contributions

**Zeweter Abebe:** conceptualization, methodology, writing – review and editing, formal analysis, supervision, investigation, data curation. **Aklesia Haileyesus:** conceptualization, data collection, data analysis, and drafting of the manuscript.

## Funding

Addis Ababa University funded the data collection.

## Conflicts of Interest

The authors declare no conflicts of interest.

## Supporting information


**Table S1:** Water absorption capacity, wet gluten content, and gluten‐to‐protein ratio of bread samples with varying levels of cauliflower leaf powder CLP.

## Data Availability

The data will be available from the corresponding author upon reasonable request.
