# Supplementary material for: Climate‐Smart Bread With Cauliflower Leaf Powder: Enhancing Nutrition and Reducing Food System Waste and Carbon Footprint, Addressing Sensory Trade‐Offs and Improvement Opportunities
Source: Food Sci Nutr. 2026 Feb 13;14(2):e71533. doi: 10.1002/fsn3.71533 (PMC12903547; doi:10.1002/fsn3.71533)
Supplement: Supplementary file 1 — Table S1: Water absorption capacity, wet gluten content, and gluten‐to‐protein ratio of bread samples with varying levels of cauliflower leaf powder CLP. [file FSN3-14-e71533-s001.docx]

**Supplementary Table S1. Water Absorption Capacity, Wet Gluten Content, and Gluten-to-Protein Ratio of Bread Samples with Varying Levels of Cauliflower** Leaf Powder **CLP.**

| No. | Sample Code | Water Absorption Capacity (mL) | Wet Gluten Content (%) | Wet Gluten to Protein Ratio |
| --- | --- | --- | --- | --- |
| 1 | F1 | 2.00 ± 0.70ᵃ | 13.60 ± 0.03ᵃ | 1.26 |
| 2 | F2 | 1.50 ± 0.70ᵃᵇ | 13.05 ± 0.12ᵃᵇᶜ | 1.25 |
| 3 | F3 | 1.50 ± 0.00ᵃᶜ | 13.28 ± 0.04ᵃᵇ | 1.28 |
| 4 | F4 | 2.25 ± 0.35ᵃᵈ | 12.68 ± 0.45ᵇᶜ | 1.30 |
| 5 | F5 | 1.75 ± 0.35ᵃ | 12.49 ± 0.43ᶜ | 1.38 |
| 6 | F6 (Control) | 1.25 ± 0.35ᵃᵉ | 12.85 ± 0.27ᵇᶜ | 1.62 |

**Note:** Values are presented as **mean ± standard deviation (n = 3)**. **Water absorption capacity is expressed as mL water/g flour, wet gluten content as %, and gluten-to-protein ratio as a dimensionless index.** Different superscript letters (**a–e**) within a column indicate **statistically significant differences among sample means (one-way ANOVA, p < 0.05)**. **CLP refers to cauliflower leaf powder incorporated as a partial replacement of wheat flour.**
